# Cross Type Neutralizing Antibodies Detected in a Unique HIV-2 Infected Individual From India

**DOI:** 10.3389/fimmu.2018.02841

**Published:** 2018-12-17

**Authors:** K. K. Vidyavijayan, Narayanaiah Cheedarala, Hemalatha Babu, Lucia K. Precilla, Pattabiraman Sathyamurthi, Padmapriyadarsini Chandrasekaran, Kailapuri G. Murugavel, Soumya Swaminathan, Srikanth P. Tripathy, Luke Elizabeth Hanna

**Affiliations:** ^1^Division of HIV/AIDS, Department of Clinical Research, National Institute for Research in Tuberculosis (ICMR), Chennai, India; ^2^Department of Infectious Diseases Laboratory, Y.R. Gaitonde Centre for AIDS Research and Education (YRG CARE), Chennai, India

**Keywords:** broadly cross neutralizing antibody, HIV-2 infection, host immunity, glycan dependent antibody, vaccine

## Abstract

**Background:** Infection with HIV-2, a retrovirus that is closely related to HIV-1, is characterized by slower disease progression and transmission, longer latency period and low or undetectable viremia. Host immunity, including production of potent neutralizing antibodies, may be one of the possible contributors to the distinction between the two infections. In an attempt to understand whether HIV-2 infection results in production of neutralizing antibodies and to characterize the nature of the neutralization response we screened plasma of 37 HIV-2 infected individuals for the presence of broadly neutralizing antibodies.

**Materials and Methods:** Thirty seven asymptomatic, ART-naïve, HIV-2 infected individuals were recruited for the study. Plasma obtained from these individuals were screened for the presence of broadly cross reactive neutralizing antibodies (BCNabs) using the standard neutralization screening protocol with a panel of HIV-1 and HIV-2 pseudoviruses. Plasma exhibiting broad neutralization activity were assessed for their potency employing a titration assay. Further, an attempt was made to characterize the neutralization specificity of the plasma exhibiting broad and potent neutralization activity.

**Result:** While majority of the samples tested were capable of neutralizing HIV-2 pseudoviruses with high to moderate potency, one unique sample demonstrated broad cross clade and cross type neutralization with ability to strongly neutralize the vast majority of both HIV-1 and HIV-2 viruses tested (19/20). Preliminary analyses indicate the possible presence of antibodies with multiple glycan epitope binding specificities.

**Conclusion:** The study identified a unique HIV-2 sample with exceptional ability to neutralize HIV-2 viruses and cross-neutralize HIV-1 viruses with great breadth and potency. This sample holds promise for isolation of novel monoclonal antibodies that may exploited as potential therapeutic tools for HIV infection.

## Introduction

One of the greatest challenges facing HIV vaccine researchers is the discovery of a vaccine that can induce the production of potent broadly neutralizing antibodies (bNAbs) that can act against a large number of contemporaneous viral strains (1). Antibodies that can neutralize diverse viral isolates are reported to be present in about 10–30% of individuals infected with HIV for 2–4 years (2, 3). One percent of these individuals in turn harbor antibodies that exhibit exceptional cross-clade neutralizing activity with high potency, and are referred to as elite neutralizers (4).

HIV-2, a closely related retrovirus to HIV-1, also causes AIDS in humans (5). Comparison of the two virus types have revealed some similarities and suggested some important differences. Both viruses share up to 60% sequence identity in their proteins, have the same modes of transmission and are associated with similar opportunistic infections resulting in AIDS (6, 7). Yet, HIV-2 infection is characterized by slower disease progression and transmissibility, longer latency period, low or undetectable plasmatic viral levels, and slower progression to AIDS than HIV-1 (7, 8). The difference in clinical outcomes between the two HIV types offers an opportunity to understand the host immune factors and viral factors responsible for natural control of the virus. Very few studies have explored bNAb response in HIV-2 infected individuals, possibly due to the very small numbers of HIV-2 infection (9, 10). The few available studies report that neutralizing antibodies elicited during HIV-2 infection are similar to or less potent than HIV-1 NAbs, and exhibit equivalent or greater breadth of reactivity against virus strains (11, 12). In an attempt to understand the reason behind the better control of HIV-2 infection, we screened the plasma of a cohort of HIV-2 infected individuals for the presence of broadly cross neutralizing antibodies.

## Materials and Methods

### Ethics Statement

The study was conducted with the approval of the institutional ethics committee of the National Institute for Research in tuberculosis (TRC IEC No: 2009009) Chennai, India as well as the IRB of GHTM, Tambaram, Chennai and YRG CARE, Chennai (YRG IRB No: 279) and written informed consent were obtained from all the study participants. All experiments were performed in accordance with relevant guidelines and regulations.

### Study Population

Ten milliliters of blood was obtained from a south Indian cohort of 37 antiretroviral therapy naïve, asymptomatic, HIV-2–infected subjects. This included 15 cases recruited from the ART clinic at the Government Hospital for Thoracic Medicine, Tambaram, Chennai, during the period 2012–2014 and 22 cases recruited from the YRG Care center, Chennai, India, during the period 2014–2016. All subjects were free of HIV-1 co- infection at the time of sample collection. Diagnosis and confirmation of HIV-2 infection was achieved using serological and molecular diagnostic tests. Serological testing was done using the Retro quick rapid test (Qualpro Diagnostics) and HIV Tridot (J. Mitra). Samples were further tested using a molecular test to confirm the absence of HIV-1 co-infection, with negative detection in HIV-1 DNA- PCR and positive detection in HIV-2 gene specific PCR. The clinical details, CD4+ T cell count and hematology profile were recorded for all the study participants. Plasma was separated and stored at −80°C. Before use, plasma samples were heat inactivated at 56°C for 1 h.

### HIV-1 and 2 Pseudovirus Production and Titration

A panel of 18 HIV-1 pseudoviruses belonging to different clades and all 3 tiers of neutralization (easy, moderate and difficult-to-neutralize viruses) were produced in 293 T cells by co-tranfection of pEnv and pSGΔEnv back bone using the standard Calcium Phosphate method and titrated on TZM-bl cells (13). Infectious stocks of HIV-2 7312A virus strains were also produced as described above. HIV-2 NIRT010 primary virus was produced by PBMC coculture. Single residue mutant pseudoviruses (DU156 N160K, DU156 N332A) were obtained as a kind gift from Dr. Lynn Morris (NICD, Johannesburg) and JRFL-D279A, JRFL E168K-N156K, JRFLW168K-N160K, and JRFLE168K-N332A and MuLV plasmids were obtained from Dr. Raghavan Varadharajan, IISC, Bangalore. Pseudoviruses belonging to HIV-1 clades A, B, C, A/E, and A/G, HIV-2 7312A, RSC3 WT, and RSC3 Δ371I/P363N recombinant proteins were obtained from the NIH AIDS Reagent Program. Neutralization potential of the plasma samples was determined based on an observed reduction in luciferase gene expression after a single round of infection in TZM-bl cells with env-pseudotyped viruses using the standard protocol (13). Murine leukemia virus was used as the control virus.

### Epitope Binding Antibody Assay

#### Linear PepScan With Overlapping gp160 Peptides

Fifteen amino acid long linear peptides with 11 amino acid overlap, spanning the entire length of gp160 of the Indian subtype C virus, 93IN101, were synthesized commercially (Infinity Biotech andResource Inc., PA). The peptides were adsorbed onto 96-well ELISA immuno maxisorp plates (Thermo Fisher) at a concentration of 5 μg/ml in 100 mM NAHCO_3_, pH 9.6, by overnight incubation at 4°C and ELISA was performed as described previously (14). Briefly each peptide was coated in individual wells of 96-well ELISA plates overnight at a concentration of 5 μg/ml. Plates were blocked for 1 h at room temperature. Plasma sample diluted 1:50 in diluent and was added to respective wells of the peptide coated 96-well plates. After incubation at 37°C for 1 h, the plates were washed four times with PBST buffer. Secondary incubation was performed in the presence of HRP-conjugated anti-human antibody. The wells were then washed with PBST four times followed by addition of 100 μl/well aqueous 3,3′,5,5′-tetramethylbenzidine substrate. Color development was stopped using 2 n sulfuric acid after 30 min. The absorbance was measured at 450 nm. Plasma samples were tested in duplicate and each experiment was performed on two independent occasions. Healthy Human Plasma pool (HHP) was included as the negative control in all experiments.

#### ELISA With Conformational Proteins

In order to determine binding reactivity of the antibodies in the plasma to whole conformational proteins, plasma were tested in an ELISA with HIV-1 subtype C (C.1086), D7gp120 monomer (Cat No.12582) and gp140C trimer (Cat.No. 12581), and its mutant forms C.1086 D7gp120K160N (12579) and C.1086 gp140C K160N (12580) as well as V1V2 tag (Cat.No. 12568), all obtained from the NIH AIDS Research Reagent Program, Division of AIDS, NIAID, NIH. The proteins were adsorbed onto 96-well ELISA immunomaxisorp plates (Thermo Fisher) at a concentration of 2 μg/mL in 100 mM NaHCO_3_, pH 9.6, by overnight incubation at 4°C and tested using the ELISA protocol described above. Plasma samples were tested in triplicate at a dilution of 1:200. HHP used as the negative control. The experiment was performed on two independent occasions and mean values were calculated.

#### ELISA With Recombinant Proteins

To identify CD4 binding site (CD4BS) antibodies, ELISA was performed with recombinant proteins, Resurfaced stabilized core protein 3 (RSC3 WT) (Cat No.12042) and RSC3Δ371I/P363N (Cat No.12362) mutant protein popularly employed for this purpose. These proteins were obtained from the NIH AIDS Reagent Program, Division of AIDS, NIAID, NIH. The recombinant proteins were adsorbed onto 96-well ELISA maxisorp plates at a concentration of 2 μg/mL in 100 mM NaHCO_3_, pH 9.6, by overnight incubation at 4°C. Samples were tested at 1:100 dilution for the identification of CD4BS antibody specificty. HHP was used as the negative control and the experiment was performed on two independent occasions.

### Virus Neutralization Assay

The neutralizing property of plasma samples was evaluated using the standard neutralization assay (13). Briefly, TZM-bl cells were infected with different pesudoviruses in the presence or absence of plasma. After 48 h, 100 μl of BriteLite (PerkinElmer) substrate was added to the wells. 100 μl of supernatant was transferred to a solid opaque plate and luminescence was measured as relative luminescence units (RLU) using the Vector 3 luminometer. Initial neutralization screening assays were performed at a plasma dilution of 1:10 dilution. Subsequently, for the determination of ID50 values, serial dilutions of the plasma samples ranging from 1:20 to 1: 43,740 or 1:10 to 1: 100,000 were used.

### Statistical Analysis

Statistical analysis was performed using the software Graphpad prism version 5.0 for determination of ID_50_ values through a dose-response curve fit with non-linear regression.

## Results

### Characteristics of the HIV-2 Infected Subjects

All 37 samples were HIV-1 negative as determined by serological and molecular methods.The study group had a mean age of 43 years (range 18–57 years) at the time of sampling; 12/37(32%) were females. The median CD4+ and CD8+ T cell counts were 618 (range 100–3,000) cells/mm^3^ and 932 (range 150–2000) cells/mm^3^. The complete demographic details of the participants are provided in Table 1.

**Table 1:** Characteristics of HIV-2 infected individuals during enrolled in the study study.

**Samples (*n* = 37)**	
Gender(% of woman)	32
Mean age(year)	43
**CD4**+ **T cells/mm**^3^	
With >500	18
200–500	13
< 200	6
**CD8**+ **T cells/ mm**^3^	
With >500	30
200–500	5
< 200	2

### Both Intratype and Intertype Neutralizing Activity Identified in one HIV-2 Infected Individual

To assess the neutralizing activity, plasma samples of all 37 individuals were tested against a 293 T cell derived HIV-2 enveloped pseudovirus HIV-2 7312A and a PBMC derived primary virus HIV-2 NIRT010. All plasma samples showed potent heterologous neutralization against both the viruses. The neutralization titers of all the plasma samples are represented in Table 2. Geometric mean titer (GMT) was calculated for each plasma against the HIV-2 viruses (Table 2). GMT of the plasma samples ranged from 80 to 23053.

**Table 2:** Neutralization titers (ID_50_) of 37 HIV-2 plasma aganist HIV-2 viruses ID_50_.

**Sample ID**	**7312A**	**HIV-2 NIRT010**	**MuLV**	**GMT**
NIRT-01	7,380	391	< 10	1,699
NIRT-02	8,185	290	< 10	1,542
NIRT-03	2,440	3,439	< 10	2,896
NIRT-04	7,320	2,295	< 10	4,098
NIRT-05	ND	ND	ND	NA
NIRT-06	18,833	5,480	< 10	10,158
NIRT-07	180	20	< 10	61
NIRT-08	4,980	2,854	< 10	3,770
NIRT-09	2,880	1,260	< 10	1,904
NIRT-10	540	594	< 10	566
NIRT-11	12,150	118	< 10	1,199
NIRT-12	7,236	8	< 10	7,236
NIRT-13	420	6,801	< 10	1,690
NIRT-14	280	2052	< 10	757
NIRT-15	180	216	< 10	197
NIRT-16	11,543	31,000	< 10	18,916
NIRT-17	2,520	900	< 10	1,505
NIRT-18	9,180	2,100	< 10	4,390
NIRT-19	10,935	1,371	< 10	3,871
NIRT-20	360	60	< 10	146
NIRT-21	36,453	14,580	< 10	23,053
NIRT-22	180	180	< 10	180
NIRT-23	12,124	12,765	< 10	12,440
NIRT-24	23,858	159	< 10	1,948
NIRT-25	14,580	510	< 10	2,726
NIRT-26	20,655	450	< 10	3,048
NIRT-27	2,520	58	< 10	384
NIRT-28	10,692	465	< 10	2,231
NIRT-29	12,180	478	< 10	2,414
NIRT-30	3,650	94	< 10	588
NIRT-31	17,921	103	< 10	1,361
NIRT-32	24,723	140	< 10	1,860
NIRT-33	120	14,580	< 10	1,322
NIRT-34	153	135	< 10	143
NIRT-35	748	91	< 10	261
NIRT-36	121	58	< 10	84
NIRT-37	250	161	< 10	200

*ID_50_ values refer to the reciprocal dilution that conferred 50% neutralization of viruses in a TZM-bl assay. Assays were done in duplicate on two independent occasions. ND, Not Done; NA, Not applicable; MuLV, murine leukemia virus control*.

We further tested all the plasma samples aganist HIV-1 pseudovirus panel in order to determine if any of the plasma samples possessed intertype (crosstype) neutralization potential. We used a panel of tier 1 HIV-1 pseudoviruses belonging to different subtypes. Interestingly it was observed that one of the 37 plasma (NIRT-06) alone was able to strongly neutralize all the 6 tier 1 pseudoviruses (>80% neutralization) while the remaining samples showed no neutralization activity. Based on the definition of Montefiori et al. (13), ≥60% neutralization was defined as strong neutralization. Encouraged by this finding, we went on to further test this unique sample with a reference panel of tier-2 and tier 3 pseudoviruses that are commonly employed for standardized assessment of neutralizing antibody response in the HIV-1 infected individuals. The plasma again showed strong neutralization of 5 of the 6 tier 2 pseudoviruses except pIndie, and 6 of the difficult to neutralize tier 3 panel including HIV-1 clade A, B and AG reference viruses (Table 3). Based on these observations, sample NIRT-06 was identified to possess broad cross type and cross-clade neutralizing (BCN) activity.

**Table 3:** Neutralization breadth of BCN plasma against HIV-1 pseudoviruses.

**Tier**	**HIV-1 Viruses**	**Subtype**	**% of Neutralization**
Tier 1	ZM197M.PB7	C	96
	SF162.LS	B	88
	242.14	AG/A1	83
	ZM109F.PB4	C	96
	6535.3	B	96
	GS015.EC	C	90
Tier 2	16936.2.21	C	95
	TRO.11	B	93
	CAP210.2.00.E8	C	76
	DU156.12	C	93
	280-5	AG/A1	92
	P INDIE	C	20
Tier 3	33.7	A1U	95
	PVO.4	B	90
	251.18	AG	94
	TRJO.4551-58	B	70
	278.50	A1U	97
	257	AG	83
Control virus	MuLv	–	8

*Cross neutralization activity of NIRT-06 plasma sample against 18 pseudoviruses belonging to tier 1, 2, and 3 from different subtypes of HIV-1 were screened. The percent of neutralization by the plasma against pseudovirus at a dilution of 1/10 is shown. Each experiment was performed at least two independent occasions. MuLV, murine leukemia virus control*.

### The Unique Cross-Clade Neutralizing Plasma Exhibits High Potency of Neutralization

**Figure 1:**
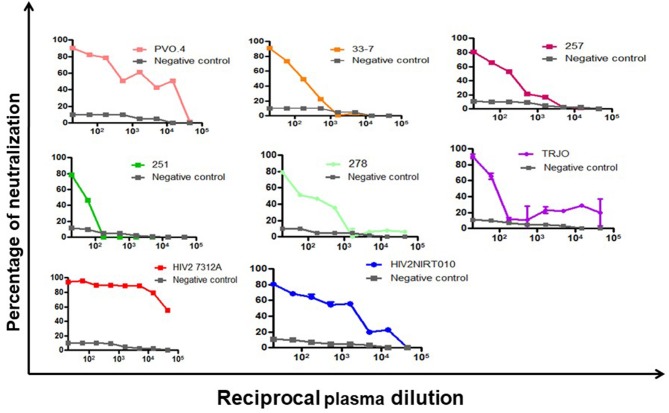
Neutralization potency of BCN plasma sample against HIV-1 pseudoviruses. Dose response curve showing the neutralization poyency with a dilution starting from 1:20 to 1:43740 against 6 tier-3 pseudoviruses in TZM-bl cells. The BCN plasma was able to neutralize all tier-3 pseudoviruses which are highly resistant to neutralization with high efficiency as seen by the high ID_50_ values.

The neutralization potency of the BCNAbs in NIRT-06 plasma was determined using the neutralization titer assay with a group of HIV-1 tier 3 pseudoviruses (Figure 1). The ID_50_ values are shown in Table 4. The plasma sample neutralized all tier 3 viruses, except 251–18 with an ID_50_ value>150. A high ID_50_ >1,000, was observed with both the subtype B pseudoviruses used in the assay, and >10,000 with the HIV-2 viruses (Table 2) indicating that the BNAbs in the plasma possess very good potency.

**Table 4:** Neutralization potency of BCN plasma antibodies.

**Sample ID**			**HIV-1 tier 3 viruses**				**HIV-2 viruses**		**Control virus**
**NIRT-06**	**Subtype-B**		**CRF02_AG**				**AB isolate**		
	PVO.4	TRJO	33-7	257-31	251-18	278-50	7312A	HIV-2 NIRT010	MuLV
	3,208	5,928	227	325	78	400	18,833	5,480	< 10

*Neutralization titers of BCN plasma against HIV-1 tier 3 pseudviruses and HIV-2 viruses. BCN plasma was able to neutralize tier-3 pseudoviruses which are highly resistant to neutralization as seen by the ID_50_ value. Assays were done in duplicate on two independent occasions. MuLV, murine leukemia virus*.

### Cross Reactivity to Linear and Conformational Epitopes of HIV-1

**Figure 2:**
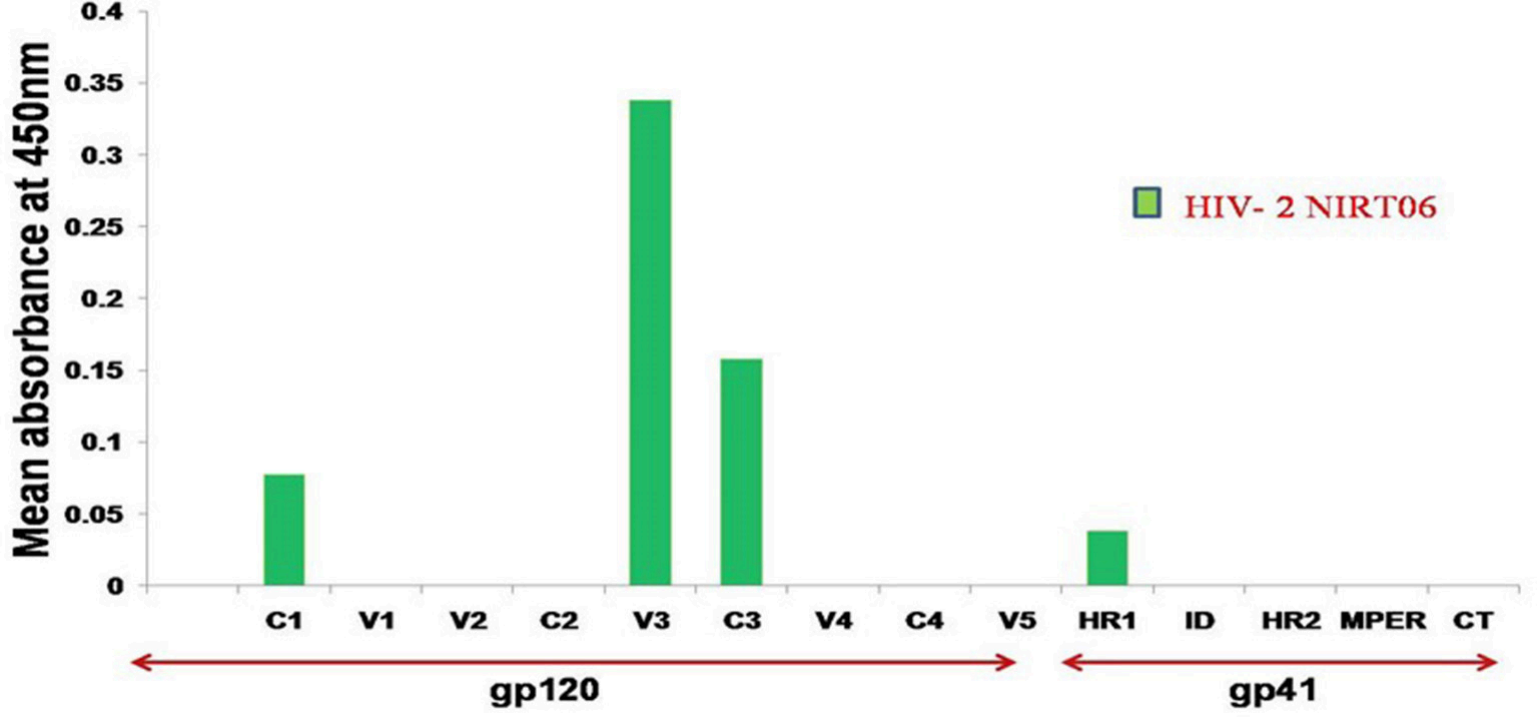
Linear PepScan analysis of BCN plasma sample. The BCN sample exhibiting potent cross type neutralization was tested at a dilution of 1:50 for the identification of binding reactivity. Healthy Human Plasma (HHP) was used as the negative control. Mean absorbance was calculated from each experiment performed in duplicate and on two independent occasions.

**Figure 3:**
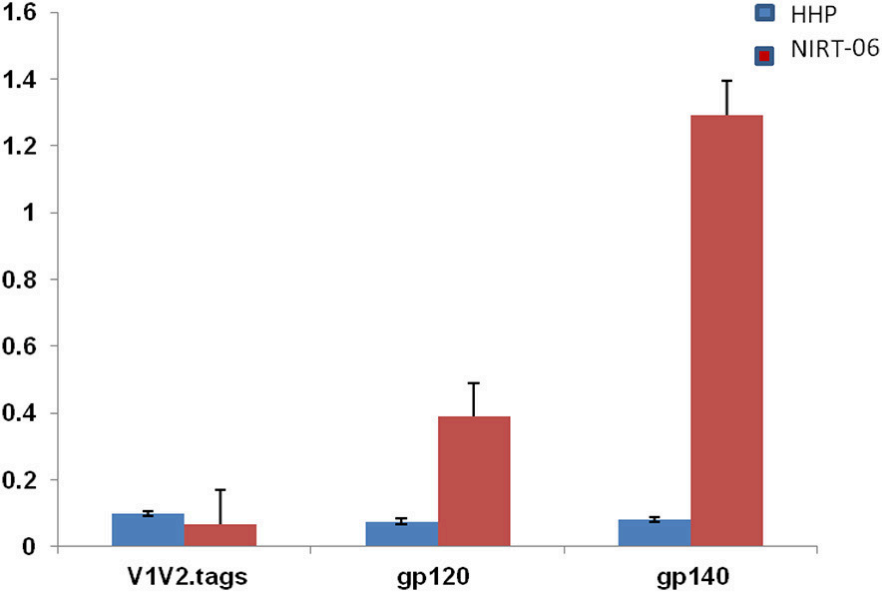
ELISA with HIV-1 (C1086) V1V2 tag, gp120 and gp 140 proteins. ELISA was done with C1086 V1V2 tag, gp120, and gp140 monomeric and trimeric proteins from HIV-1 subtype C. Mean absorbance was calculated from each experiment performed in duplicates and two independent experiments.

To characterize the neutralization specificity of BCNAbs in the plasma, we performed ELISA with linear peptides and conformational proteins of HIV-1. Due to the non-availability of HIV-2 peptides ELISA was performed using HIV-1 peptides and proteins. The linear pepscan analysis showed good immunoreactivity to the third variable (V3) and fourth constant (C4) region peptides and weak reactivity to C1 and HR1 peptides (Figure 2). There was no reactivity to any of the cytoplasmic tail peptides. The sample was then tested in an ELISA with HIV-1 subtype C V1–V2 tag and Env subunit protein C.1086 D7gp120 and C.1086 gp140C. A better binding reactivity was observed with the gp140 trimeric protein than with the gp120 monomeric protein (Figure 3).

### Evidence for N Linked Glycan Dependent Neutralizing Activity in the BCN Plasma

**Figure 4:**
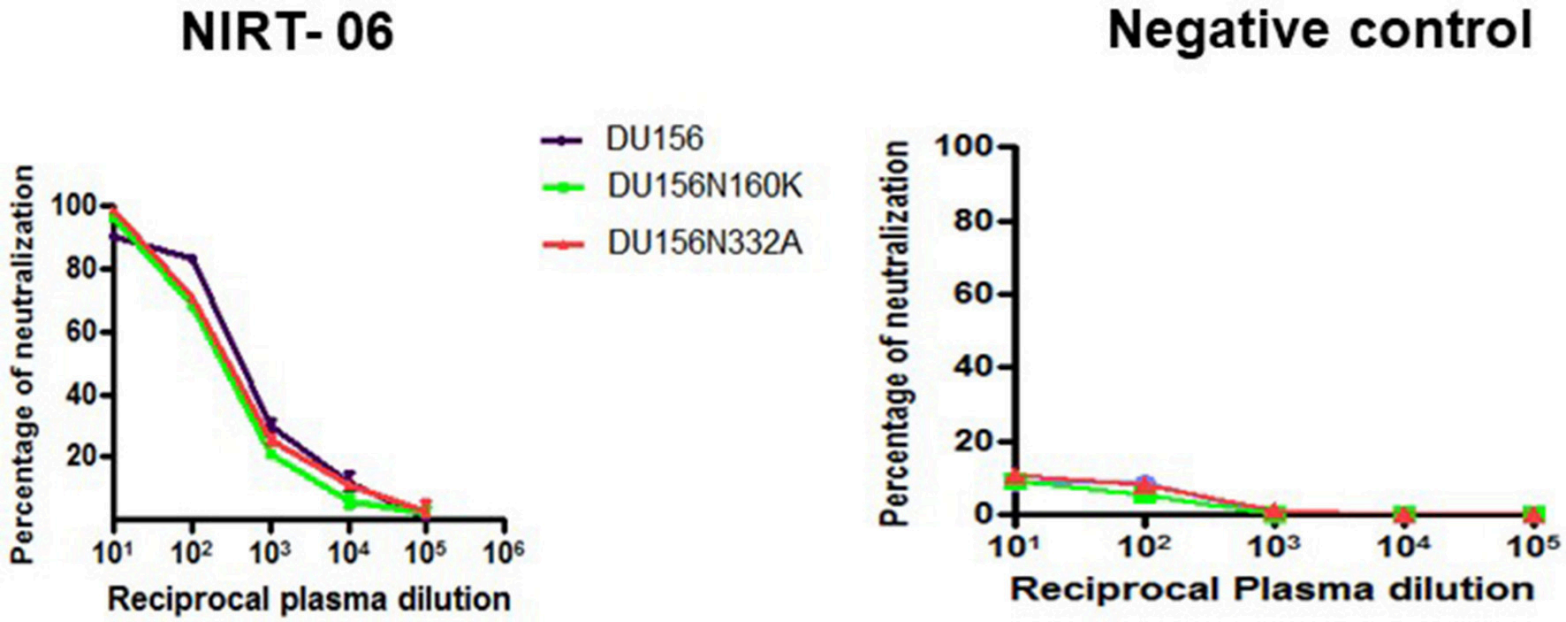
Identification of Glycan dependent antibodies. Neutralization curves of BCN plasma sample aganist HIV-1 subtype C tier-2 pseudoviruses DU156 WT and mutant with substitutions at glycan position 160 and 332. Neuralization titration assay was performed with BCN plasma sample in duplicates at dilutions ranging from 1:10 to 1:100,000 on two independent occasions.

**Figure 5:**
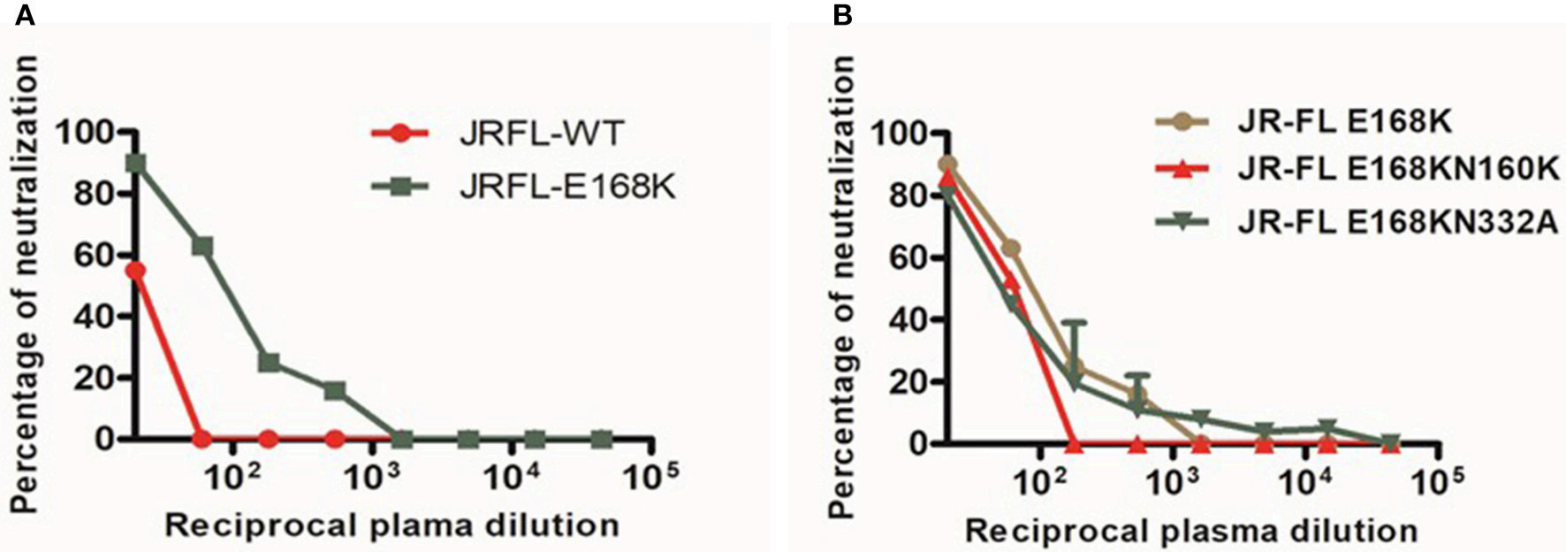
Neutralization sensitivity assay to detect Glycan-dependent antibodies. **(A)** Dose response curve of BCN plasma with JR-FL wild type (WT) and E168K mutant for recognzing glycan depend antibodies. **(B)** Neutralization activity against glycan positions at N156 and N160 in the JR-FL E168K mutated strain for the confirmation of glycan dependent bNAbs.

In order to screen for glycan dependent neutralizing activity, neutralization assay was performed with wild type and glycan mutant pseudoviruses, HIV-1 subtype C DU156 and its mutants DU156N160K, widely employed for the identification of V1/V2 glycan specific NAbs (PG9/16) in HIV infected individuals. The PG9/16 class of antibodies are dependent on an N-linked glycan at position 160 in the V1/V2 region for neutralization of HIV. A significant decrease in neutralization sensitivity to the order of 3-fold or more in ID_50_ value for the mutant DU156 as compared to the wild type is generally considered as an indication for the presence of PG9/PG16 –like antibodies in the sample. NIRT-06 plasma exhibited strong neutralization activity against DU156WT (Figure 4, Table 5) with a 1.6-fold decrease in neutralization of the DU156N160K mutant virus, suggesting the possible presence of PG9/PG16 like antibodies in the BCN sample. In order to further confirm the N160 glycan dependent nature of the antibodies in NIRT-06, the sample was tested again in a neutralization assay with JR -FL WT and JR-FL E168K single mutant, and JR-FL E168K N156K and JR-FLE168K N160K double mutants. Presence of glutamic acid (E) at position 168 in the subtype B JR-FL WT strain makes it resistant to neutralization by PG9/PG16 like bNAbs. In the single mutant, JR-FL E168K, E is replaced with lysine (K) making it sensitive to neutralization by this class of bNAbs. NIRT-06 sample neutralized the single mutant pseudovirus better than the JR-FL WT virus (Figure 5A and Table 6a) and JR-FL E168K N156K and JR-FL E168K N160K double mutants showed greater reduction in neutralization as compared to single mutant (Figure 5B and Table 6b).

**Table 5:** Identification of glycan dependent antibodies in BCN sample.

**Sample ID**	**ID50**			**Fold change**	
	**WT**	**N160K**	**N332A**	**N160K**	**N332A**
NIRT-06	732	469	495	1.6	1.5
Negative control	< 5	< 5	< 5	NA	NA

*Neutralization activity aganist DU156 WT and mutants for identifying glycan dependent antibodies. Fold change calculated as ID_50_ of HIV-1DU156WT/ID_50_ of HIV-1DU156 mutant*.

**Table 6a:** Neutralization activity of the BCN plasma sample against HIV-1 JR-FL and E168K mutant pseudoviruses.

**Sample ID**	**ID**_**50**_		**Fold change**
	**WT**	**E168K**	**E168K**
NIRT-06	40	116	2.92
Negative control	< 5	< 5	NA

*The fold change calculated by ID_50_ of JR-FL E168K/ID_50_ of JR-FL WT. NA, Not applicable*.

**Table 6b:** Neutralization activity against HIV-1 JR-FL E168K and double mutated pseudoviruses JR-FL E168K N156K JR-FL E168K N160K and JR-FL E168K N332A.

**Sample ID**	**ID**_**50**_			**Fold change**	
	**JR-FL E168K**	**N160K**	**N332A**	**N160K**	**N332A**
NIRT06	116	82	83	1.4	1.39
Negative control	< 5	< 5	< 5	NA	NA

*The fold change calculated by ID_50_ of JR-FL double mutant/ID_50_ of JR-FL single mutant. NA, Not applicable*.

NIRT-06 plasma was also tested for V3 glycan dependent neutralization activity using the DU156 and DU156N332A pseudoviruses. There was a 1.5-fold decrease in neutralization of the single mutant than the wild type virus (Figure 4 and Table 5). The PGT series of neutralizing antibodies are dependent on the presence of an N- glycan at position 332 in the V3 region of the Env, and can be identified using these viruses. Similar observations were made with JR-FL WT and JR-FL N332A pseudoviruses confirming the possible presence of PGT-like antibodies in the NIRT-06 plasma (Figure 5B and Table 6b).

### No Evidence for CD4- Binding Site and MPER Specific Antibody Response

Comparison of the binding response of NIRT-06 plasma to the recombinant CD4 BS protein, RSC3 gp120 wild type and its mutant form RSC3Δ371I/P363N, which contains a double mutation that abrogates binding of VRC01-like CD4 binding site, antibodies, showed no significant difference in binding reactivity indicating that VRC01-like CD4 binding specific Abs are not likely to be present in this sample (result not shown). The pepscan ELISA revealed that the sample did not react with any of the MPER peptides, indicating the absence of MPER specific class of antibodies in the plasma.

## Discussion

Identification of newer and better bNAbs that can protect against HIV infection is a research priority. As an important human model for exploring the features required for natural containment of a potentially lethal virus, HIV-2 infection serve as a good resource for identifying protective BNAbs as well as immunogens critical for eliciting a robust neutralization response. The present study screened a cohort of HIV-2 infected individuals for the presence of broad and potent cross type and cross clade neutralizing activity. Thirty-seven ART naive, asymptomatic, HIV-2 infected individuals from a South Indian HIV cohort were screened for the presence of bNAbs in their plasma using the standard neutralization assay. Very interestingly only one of the 37 samples tested demonstrated a remarkable breadth and potency of neutralization against a panel of HIV-1 and HIV-2 viruses, whereas the other samples only neutralized HIV-2 isolates and not any of the HIV-1 strains. Previous studies have asserted that samples with moderate breadth of neutralization against tier 2 viruses can serve as good sources for BNAbs (15, 16). This sample, NIRT-06 demonstrated a very good breadth of neutralization against tier 1, 2, and 3 HIV-1 viruses as well as HIV-2 isolates. We went on to determine the potency of neutralization of this sample by its ID_50_ titer against six difficult-to-neutralize tier-3 pseudoviruses. The plasma showed moderate potency against 3 of the tier 3 viruses (50–500), and very high potency (>1,000) against two viruses.

Encouraged by these findings, we went on to further characterize the nature of the neutralization response seen in this unique BCN plasma. We first tested the plasma in a PepScan ELISA with linear peptides spanning the entire envelope of HIV-1. Only HIV-1 peptides and proteins were employed for all binding assays, due to the non-avaliability of corresponding reagents for HIV-2. Further, the cross-reactivity of the neutralization response of the sample seen across the two HIV types prompted us to presume that the antibodies in this sample most possibly target conserved epitopes in both HIV types thus justifying the use of HIV-1 peptides as an alternative. The sample showed immunoreactivity to linear epitopes in the variable region 3 (V3) and constant region 4 (C4) of the HIV-1 envelope. In general antibodies to the V3 region are induced by most vaccine candidates; these antibodies are also known to develop early during natural infection (17, 18). However, V3 binding antibodies are known to rarely neutralize tier 2 or tier 3 psedoviruses, and are mostly only potent against tier 1 pseudoviruses. Hence these antibodies are generally considered as non-neutralizing in nature and not capable of blocking HIV infection (17).

Most frequently, conformational epitopes on the functional envelope spikes of HIV have been identified as important targets for antibodies that are neutralizing in nature. Recombinant gp140, the ectodomain of trimeric gp160, mimics the native state of the envelope spike and has therefore been employed widely to identify conformational epitope specificities (19–21). We found better reactivity with gp140 trimer than gp120 monomeric protein as well as V1V2 tag when NIRT-06 plasma was tested in an ELISA with all the three recombinant proteins. This observation suggests the possible presence of antibodies targeting conformational epitopes present in the native form of the HIV envelope protein.

BNAbs target four dominant epitopes on the HIV envelope; namely glycopeptide epitopes in the second variable loop (V2) and the N160 glycan present at the V2 apex, N332 glycan present in the third variable loop, MPER and CD4 binding site (22–28). Observation made from the initial Pepscan analysis and the non-significant interaction observed with RSC3 and mutant RSC3Δ371I/P363N proteins suggest the absence of MPER and CD4-binding site specificities in NIRT-06 (29). Although one the previous studies reported that most HIV-2 strains share a common epitope in the MPER region of gp41 with HIV-1, such that HIV-2 viruses are neutralized by the HIV-1 elicited monoclonal antibody, 4E10 (30), we did not find an antibody response to the MPER peptides in our pepscan analysis. In order to investigate the presence of glycan dependent neutralization activity, neutralization assay was performed with DU156WT, DU156N160K, and DU156N332A pseudoviruses since N-linked glycans at positions 160 and 332 are known to be vulnerable sites on the HIV envelope for the binding of glycan-dependent antibodies, such as the PG9/PG16 and PGT series of antibodies (31, 32). We observed a reduction in the levels of neutralization with mutant virus DU156N160K than wild type DU156virus suggesting the possible presence of N160 glycan dependent antibodies in this sample. Although the reduction in neutralization activity between the wild type and mutant viruses was < 3-fold, the observed reduction may still be considered significant given the fact that HIV-1 pseudoviruses were used for the characterization of the neutralization specificity, instead of HIV-2 viruses. The possible presence of N160 glycan dependant neutralization activity of the sample was further defined using wild type JR-FL and mutant JR-FL E168K pseudovirus. Stronger neutralization of the mutant than the wild type virus provides confirmatory evidence for the presence of this class of BNAbs in the plasma. In the double mutants, JR-FL E168K N156K and JR-FLE168K N160K, presence of glutamic acid (E) at position 168 masks the exposure of asparagine (N) glycans at positions 156 and 160 and thereby abrogating the binding of PG9/PG16 like antibodies. The observed reduction in neutralization of the double mutant JR-FLE168K N160K as compared to the single mutant provided further evidence to believe that the plasma contains N160-glycan dependent neutralizing antibodies (31).

The key residues of another site of vulnerability on the HIV Env is the high-mannose glycan at position N332 in the V3 loop. To identify the possible presence of N332 glycan-dependant antibodies in the sample, the plasma was tested in a neutralization assay with DU156WT and DU156 N332A mutant pseudoviruses. The reduced level of neutralization of the mutant virus as compared to the wild type virus suggest that the BCN sample may also contain N332 specific NAbs. The PGT (PGT 121-like and PGT128-like) series of antibodies are those that bind to the N332 glycan site and bring about virus neutralization (32–34). The presence of PGT type of antibodies in the plasma was further confirmed by repeating the neutralization analysis with JRFL and its mutant pseudovirus JR-FL E168K N 332A (28). The observed reduction in neutralization of the mutant as compared to wild type virus, authenticates the presence of N332 glycan dependent neutralizing antibodies in the NIRT- 06 plasma.

A vaccine that aims to elicit strong HIV neutralizing antibodies that can protect against HIV-1 infection must be able to overcome the genetic variability of HIV strains (35). This study has identified a very unique sample with a potent broadly cross reactive neutralizing antibody responses capable of neutralize both HIV-1 and HIV-2 isolates belonging to different clades. The present study however has not determined whether the observed cross type neutralizing activity seen in this sample is monoclonal or polyclonal in nature. However, to our knowledge this is the first study to identify second generation of broad and potent neutralizing Abs from a HIV-2 infected individual that can cross neutralize HIV-1 as well as HIV-2 viruses. Plasma with cross clade and cross type Nab response with multiple epitope specificities as well as great breadth and potency of neutralization is certainly of particular interest for detailed investigation in the context vaccine design, thus warranting additional studies which are currently on-going in our laboratory. Isolation of monoclonal antibodies from this sample would be of immense value for use as therapeutic tools.

## Author Contributions

KV, LH, ST, and SS designed the study. PC, KM, LP, and PS helped in sample collection. KV, NC, and HB participated in sample processing and data analysis. KV drafted the paper.

### Conflict of Interest Statement

The authors declare that the research was conducted in the absence of any commercial or financial relationships that could be construed as a potential conflict of interest.
